# Validation of World Health Organisation HIV/AIDS Clinical Staging in Predicting Initiation of Antiretroviral Therapy and Clinical Predictors of Low CD4 Cell Count in Uganda

**DOI:** 10.1371/journal.pone.0019089

**Published:** 2011-05-12

**Authors:** Steven Baveewo, Francis Ssali, Charles Karamagi, Joan N. Kalyango, Judith A. Hahn, Kenneth Ekoru, Peter Mugyenyi, Elly Katabira

**Affiliations:** 1 Clinical epidemiology unit, College of Health Sciences, Makerere University Kampala, Kampala, Uganda; 2 Department of Pharmacy, College of Health Sciences, Makerere University Kampala, Kampala, Uganda; 3 Department of Medicine, College of Health Sciences, Makerere University, Kampala, Uganda; 4 Joint Clinical Research Centre Kampala, Kampala, Uganda; 5 University of California San Francisco, San Francisco General Hospital, San Francisco, California, United States of America; 6 Makerere University - University of California Los Angeles - University of California San Francisco VCT research collaboration, Kampala, Uganda; University of Liverpool, United Kingdom

## Abstract

**Introduction:**

The WHO clinical guidelines for HIV/AIDS are widely used in resource limited settings to represent the gold standard of CD4 counts for antiviral therapy initiation. The utility of the WHO-defined stage 1 and 2 clinical factors used in WHO HIV/AIDS clinical staging in predicting low CD4 cell count has not been established in Uganda. Although the WHO staging has shown low sensitivity for predicting CD4<200cells/mm^3^, it has not been evaluated at for CD4 cut-offs of <250cells/mm^3^ or <350 cells/mm^3^.

**Objective:**

To validate the World Health Organisation HIV/AIDS clinical staging in predicting initiation of antiretroviral therapy in a low-resource setting and to determine the clinical predictors of low CD4 cell count in Uganda.

**Results:**

Data was collected on 395 participants from the Joint Clinical Research Centre, of whom 242 (61.3%) were classified as in stages 1 and 2 and 262 (68%) were females. Participants had a mean age of 36.8 years (SD 8.5). We found a significant inverse correlation between the CD4 lymphocyte count and WHO clinical stages. The sensitivity the WHO clinical staging at CD4 cell count of 250 cells/mm^3^ and 350cells/mm^3^ was 53.5% and 49.1% respectively. Angular cheilitis, papular pruritic eruptions and recurrent upper respiratory tract infections were found to be significant predictors of low CD4 cell count among participants in WHO stage 1 and 2.

**Conclusion:**

The WHO HIV/AIDS clinical staging guidelines have a low sensitivity and about half of the participants in stages 1 and 2 would be eligible for ART initiation if they had been tested for CD4 count. Angular cheilitis and papular pruritic eruptions and recurrent upper respiratory tract infections may be used, in addition to the WHO staging, to improve sensitivity in the interim, as access to CD4 machines increases in Uganda.

## Introduction

There have been significant declines in HIV-related morbidity and mortality since the advent of anti-retroviral therapy (ART) [1], [2] Initiation of ART is based on CD4 cell count or being classified as being in World Health Organisation (WHO) HIV/AIDS clinical stage III or IV [2]. However, although the CD4 cell counts testing is critical in the determination of eligibility for ART, many HIV treatment centres in resource limited settings lack CD4 testing facilities. In the absence of CD4 testing in rural primary health care facilities, WHO HIV/AIDS clinical staging is used to recommend when to initiate ART [2], [3].

Some previous studies have explored the utility of using other predictors of low CD4 cell count to guide initiation of antiretroviral therapy. Other clinical factors, such as anaemia and body mass index have low sensitivities in detecting eligibility for HAART initiation [4] while other studies have shown that clinical factors such as anaemia could double sensitivity if used together with the WHO clinical staging guidelines [5]. Total lymphocyte counts <1200 cells/microl used with WHO staging showed good specificity (>99%) [5], and a good correlation between total lymphocyte count and CD4 cell count in a Ugandan study [6].

In Uganda, although the availability of CD4 testing is increasing, its access is limited due to cost and a lack of laboratory infrastructure, particularly in rural health facilities. Specifically, CD4 testing is not available in decentralized health units that include district hospitals and health centre IV facilitiesthat serve the largest population of HIV-infected Ugandans.

The WHO clinical staging system for HIV/AIDS was developed in 1990 and revised in 2007. It uses clinical parameters to classify subjects into any one of four categories i.e. stage 1 to IV, progressing from primary HIV infection to advanced HIV/AIDS. It is these categories that are used to guide decision making for the management of HIV/AIDS patients where there is limited access to laboratory services [7]. These clinical guidelines are readily available, convenient to the patient, cheap and can be applied by trained clinicians even in the most remote health facilities.

Initially the WHO clinical staging for HIV/AIDS was used in line with CD4 cut-offs of 200 cells/mm^3^ to make decisions on ART initiation. However, in Uganda the CD4 cut-off for ART initiation changed from 200 cells/mm^3^ to 250 cells/mm^3^ or 350 cells/mm^3^ for the World health organisation. Previous studies of the utility of the WHO HIV/AIDS clinical guidelines for determining ART eligibility, using the cut-off of <200 cells/mm^3^ have found the sensitivity to be in the range of 51–52% and the specificity to be in the range 68–88% [5], [8], [9]. In a Ugandan study, the sensitivity was 51% and the specificity was 88% [8].

Despite the wide use of the WHO HIV/AIDS clinical guidelines in Ugandan primary health care facilities, they have not yet been evaluated in Uganda against the new CD4 cut-offs for ART eligibility of <250cells/mm^3^ and <350 cells/mm^3^. Evaluation of the WHO HIV/AIDS staging guidelines in comparison to the newer CD4 cell count cut-offs for ART eligibility is needed to determine the potential level of misclassification. In addition, examination of the individual clinical components of the WHO HIV disease staging is needed because some clinical factors associated with stage I or II illness may be predictive of more advanced immune suppression in Uganda. We postulate that if there are significant clinical factors in stages I and II that are predictive of low CD4 counts, they could improve identification of ART-eligible subjects, thereby reducing the missed opportunities for timely ART initiation. The goal of this study was therefore to determine the diagnostic properties (sensitivity, specificity, positive predictive values and negative predictive values) of the WHO HIV/AIDS clinical staging guidelines at CD4<250cells/mm^3^ and <350cells/mm^3^ and to determine the WHO HIV/AIDS Stage I and II clinical factors (symptoms and signs) that are predictive of CD4<250cells/mm^3^ and CD4<350 cells/mm^3^.

## Methods

### Study area and subjects selection

To address the study objectives, we conducted a multi-centre, cross-sectional study from January to April 2007 at three JCRC sites located at Mengo (in Kampala, the capital city of Uganda), Jinja (85 kilometres from Kampala) and Kasana (45 kilometres from Kampala). All the three sites offered free HIV/AIDS treatment and care at the time of study recruitment. The study sites were selected for convenience. The number of subjects enrolled from each site was proportional to the number of ARV treatment naïve HIV patients seen at each study site in the previous 3 months. Subjects aged ≥18, known HIV positive, ART treatment naïve, and consented to participate in the study were consecutively enrolled to participate in the study.

### Data Collection Procedures

Clinicians with training in HIV/AIDS clinical staging administered structured questionnaires to study participants. They recorded the socio-demographic data (age, sex, marital status and occupation), and clinical factors used for the WHO HIV/AIDS staging guidelines. The latter were used these to determine the patients' clinical stage [2]. Blood samples for laboratory analysis at the Joint Clinical Research Centre (JCRC) were collected after clinical staging. Complete blood count (CBC), total lymphocyte count and sputum results were used to guide the diagnosis of neutropenia, leucopenia, anaemia and presence of pulmonary tuberculosis. The need for a CBC and/or total lymphocyte count was determined by the attending physician. The laboratory staff that handled the specimens was blinded to both the patients' identities and their HIV/AIDS clinical stage. The CBC was determined by Coulter AC^*^T 5 Diff CP, while the CD4 cell count was assessed using TriTEST CD4 FITC/CD8PE/CD3 PerCP (TRUCOUNT) reagent method. Quality assurance procedures are routinely conducted to compare the results to that of external laboratories (the National Health Laboratory Services in South Africa and to the UK Neqas in the United Kingdom).

### Sample size and Statistical Analysis

The minimum sample size of 385 subjects for the study was determined using the cross sectional studies with 4Z_α_^2^P(1−P)÷W^2^ where W is the width of confidence intervals was 1%, Z_α_ at 95% confidence interval = 1.96, with an estimate of 50% of subjects that were expected to be eligible for ART [10].

We determined the diagnostic properties of the WHO clinical staging compared to CD4<250cells/mm^3^ and <350 cells/mm^3^ and calculated 95% confidence intervals for proportions, using STATA version 10.0 (STATA Corp, College Station, TX, USA). We estimated the association between CD4<250cells/mm^3^ and <350 cells/mm^3^ and stages I and II clinical features using odds ratios at 95% confidence intervals. Variables with odds ratios and p≤0.2 at bi-variate analysis were included in a multivariate logistic regression model to determine the adjusted odds ratios for the association between clinical stages I & II clinical factors and low CD4 counts.

### Ethical considerations

Ethical approval was obtained from the Faculty of Medicine Research and Ethics Committee of Makerere University, the Joint Clinical Research Centre, and the Uganda National Council of Science and Technology. Written informed consent was obtained from each subject before enrolment into the study.

## Results

### Characteristics of the study population

Between January and April 2007 a total of 417 known HIV patients were screened. Twenty two (5.3%) participants were excluded from the study due to; age <18 years (n = 1), prior ART experience (n = 2) and inaccessible CD4 results (n = 19). A total of 395 participants were enrolled into the study. The subjects were enrolled from JCRC Mengo (75%), Jinja (14%) and Kasana (11%). The majority (61%) of the 395 subjects were classified as in clinical stages 1 and 2. Over two thirds, (68%) of all enrolled subjects were females. (Table 1). Three hundred and sixty five (92%) had attained primary level education and 117 (29.6%) were in the reproductive age group (18–49 years).

**Table 1:** Distribution of 395 participants at the CD4 cut-offs of 250cells/mm^3^ and 350cells/mm^3^ by WHO HIV/AIDS clinical stages, and sex.

WHO clinical stage	CD4 cell count cut-offs				TotalN (%)
	CD4≤250n (%)	CD4>250n (%)	CD4≤350n (%)	CD>350n (%)	
I	17 (7.5)	49 (29.0	32 (11.4)	34 (29.8)	66(16.7)
II	88 (38.9)	88(52.1)	111(39.5)	65 (57.0)	176(44.6)
III	73(32.3)	23(13.6)	83 (29.5)	13 (11.4)	96(24.3)
IV	48(21.2)	9 (5.3)	55(19.6)	2(1.8)	57(14.4)
**Total**	**226 (100)**	**169 (100)**	**281(100)**	**114(100)**	**395(100)**
**Sex**					
Male	87(38.5)	40(23.7)	104(37.0)	23(20.2)	127(32.2)
Female	139(61.5)	129(76.3)	177 (63.0)	91(79.8)	268(67.8)
**Total (%)**	**226 (100)**	**169 (100)**	**281(100)**	**114(100)**	**395(100)**

**Figure 1:**
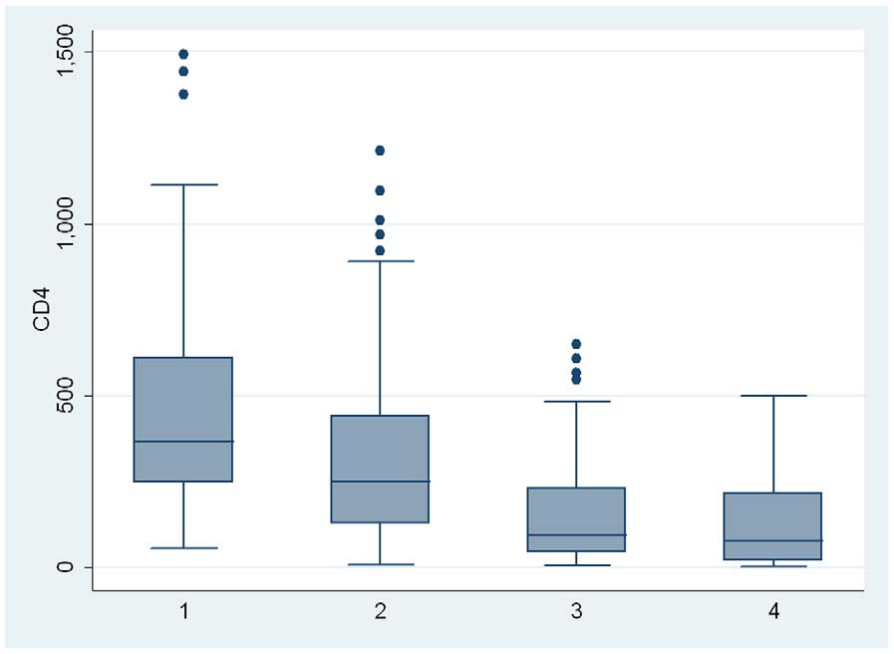
The Box plots showing the median and Inter quartile ranges CD4 cell counts of 395 participants by the WHO HIV/AIDS clinical stages I–IV.

The median CD4 cell count of those in WHO HIV/AIDS clinical stages 1 and 2 was significantly higher than that of participants in clinical stages 3 and 4. (Figure 1) We found a highly significant but moderately strong correlation between the CD4 lymphocyte count and WHO clinical stage (spearman correlation = −0.498, p<0.01). Female participants had a significantly higher mean CD4 cell count 307.23 cells/mm^3^ (SD = 270.93) as compared to male participants with 216.77 cells/mm^3^ (SD = 226.08), (p<0.0012), while the mean CD4 counts among the urban-based population was higher at 301.62 cells/mm^3^ (SD = 260.57) compared to the rural-based population with CD4 cell count of 206.03 cells/mm^3^ (248.12), p<0.0016.

### Diagnostic properties of the WHO HIV/AIDS clinical staging guidelines

The sensitivities of the WHO clinical staging at CD4 cell count cut-offs of 250 cells/mm^3^ and 350 cells/mm^3^ were 53.5% and 49.1%, while the specificities were 81.1% and 86.8% at CD4 counts of 250cells/mm^3^ and 350 cells/mm^3^. The positive predictive values at CD4 cell count of 250cells/mm^3^ and 350cells/mm^3^ was 79.1% and 90.2%, and the negative predictive values were 56.6% and 40.9%, respectively. (Table 2) There were no significant differences in the sensitivities and specificities of the WHO HIV/AIDS clinical staging guidelines at the different cut offs (CD4<250 cells/mm^3^ and CD4<350cells/mm^3^).

**Table 2:** Diagnostic characteristics of the WHO HIV/AIDS clinical staging guidelines at the CD4 cell count of less than 250 cells/mm^3^ and 350 cells/mm^3^ among 395 participants in JCRC Mengo, Kakira and Kasana health centres, Uganda.

Diagnostic characteristics	Values at CD4<250cells/mm^3^(95% CI)	Values at CD4<350cells/mm^3^(95% CI)
Sensitivity	53.5% (47–60)	49.1% (43–55)
Specificity	81.1% (75–87)	86.8% (81–93)
Positive Predictive Value	79.1% (73–86)	90.2% (85–95)
Negative Predictive Value	56.6% (50–63)	40.9% (35–47)
False Positive	18.9% (13–25)	13.2% (07–19)
False Negative	46.5% (40–53)	50.9% (45–57)

### Stage II WHO HIV/AIDS Clinical predictors of low CD4 count

Angular cheilitis and papular pruritic eruptions (Table 3) were found to be significant predictors of CD4 cell count <250cells/mm^3^ while angular cheilitis and recurrent upper respiratory tract infections (Table 4) were found to be significant predictors of CD4<350cells/mm^3^ in multivariate analysis. Angular cheilitis was defined as having splits or cracks on lips at the corner of the mouth with possible de-pigmentation, or having been successfully treated with an antifungal for angular cheilitis. Papular pruritic eruptions were defined as papular pruritic lesions, often with marked post inflammatory skin pigmentation; Recurrent upper respiratory tract infections were defined as current episode of upper respiratory tract infection with at least one episode in the past 6 months [11].

**Table 3:** Association between CD4 cell count <250cells/mm^3^ and the clinical features of the WHO HIV/AIDS clinical Stages I and II for the 242 participants in stages 1 and 2.

Clinical features	CD4<250 cells/mm^3^N (%)	CD4≥250 cells/mm^3^N (%)	Unadjusted OR(95% CI)	P value	Adjusted OR[95% CI]	P value
**WHO Stage I**						
**Asymptomatic**						
Yes	17 (25.4)	50 (74.6)	0.34 (0.17-0.64)	< 0.001	0.66(0.32-1.37)	0.27
No	88 (50.3)	87 (49.7)	1		1	
**PGL**						
Yes	2	6	0.42(0.08-2.16)	0.29		
No	103	131	1			
**WHO Stage II**						
**Recurrent URTI**						
Yes	41(58.6)	29 (41.4)	2.38(1.34-4.26)	0.0024	1.6(0.84-3.08)	0.15
No	64(37.2)	108 (62.8)	1		1	
**Moderate weight loss**						
Yes	49(47.6)	54 (52.4)	1.34 (0.80-2.25)	0.259		
No	56 (40.3)	83(59.7)	1			
**Herpes zoster**						
Yes	22(43.1)	29 (56.9)	0.98(0.52-1.84)	0.97		
No	83 (43.5)	108 (56.5)	1			
**Angular cheilitis**						
**Yes**	**20(83.3)**	**4(16.7)**	**7.8(2.47-24.75)**	**<0.000**	**4.4(1.37-14.1)**	**0.01**
**No**	**85(39.0)**	**133(61.0)**	**1**		**1**	
**Recurrent oral ulcerations**						
Yes	21(75.0)	7(25.0)	4.64(1.84-11.71)	<0.000	2.4 (0.88-6.28)	0.09
No	84(39.3)	130(60.7)	1			
**Pruritic eruption**						
**Yes**	**31(64.6)**	**17(35.4)**	**2.95(1.50-5.81)**	**0.001**	**2.1(1.02-4.34)**	**0.04**
**No**	**74 (38.1)**	**120(61.9)**	**1**		**1**	
**Seborrhoic dermatitis**						
Yes	6(60.0)	4(40.0)	2.02 (0.55-7.38)	0.280		
No	99 (42.7)	133(57.3)	1			
**Fungal nail infections**						
Yes	0(0)	1(100.0)	0.00	0.3813		
No	105 (43.6)	136 (56.4)	1			

**Table 4:** Association between CD4 <350cells/mm^3^ and the Clinical Features of the WHO HIV/AIDS clinical stages I & II among the 242 participants in Stages 1 and 2.

Clinical features	CD4<350 cells/mm^3^N (%)	CD4≥350 cells/mm^3^N (%)	Unadjusted OR(95% CI)	P value		Adjusted OR(95% CI)	P value
**WHO Stage I**							
**Asymptomatic**							
Yes	31(46.3)	36(57.3)	0.48(0.27-0.86)		0.012	1.05(0.53-2.06)	0.89
No	112 (64.0)	63(36.0)	1			1	
**PGL**							
Yes	4	4	0.68(0.17-2.81)		0.60		
No	139	95	1				
**WHO Stage II**							
**Recurrent bacterial URTI**							
Yes	**53(75.7)**	**17(24.3)**	**2.84(1.49-5.38)**		**<0.000**	**2.48(1.23-5.0)**	**0.01**
No	**90(52.3)**	**82(47.7)**	**1**			**1**	
**Moderate weight loss**							
Yes	65(63.1)	38(36.9)	1.33(0.79-2.26)		0.28		
No	78(56.1)	61(43.9)	1				
**Herpes zoster**							
Yes	29 (56.9)	22 (43.1)	0.89 (0.48-1.67)		0.72		
No	114(59.7)	77 (40.3)	1				
**Angular cheilitis**							
Yes	**22 (91.7)**	**2 (8.3)**	**8.82(1.94-39.99)**		**0.0006**	**5.82(1.27-26.8)**	**0.02**
No	**121(55.5)**	**97(44.5)**	**1**			**1**	
**Recurrent oral ulcerations**							
Yes	23 (82.1)	5(17.9)	3.60(1.29-10.0)		0.0085	2.08(0.69-6.22)	0.19
No	120 (56.1)	94(42.9)	1			1	
**Pruritic eruption**							
Yes	35(72.9)	13 (27.1)	2.14(1.06-4.33)		0.03	1.56(0.72-3.39)	0.26
No	108 (55.7)	86 (44.3)	1			1	
**Seborrhoic dermatitis**							
Yes	8 (80.0)	2(20.0)	2.87 (0.59- 13.9)		0.17	3.83(076-19.3)	0.10
No	135(58.2)	97 (41.8)	1			1	
**Fungal nail infections**							
Yes	1 (83.3)	0 (16.7)	infinite		0.41		
No	142 (70.9)	99 (29.1)					

### The effect of the clinical predictors of low CD4 cell count on sensitivity of the WHO HIV/AIDS clinical staging guidelines

To assess the effect of the use of angular cheilitis and papular pruritic eruption on the sensitivity, specificity and false negative rate to predict CD4<250cells/mm^3^ among patients in Stage I and II, a variable “clinical factor present” was generated if any of the patients in Stage II disease had one or both clinical factors of angular cheilitis and pruritic eruption present. Forty-four subjects out of the 63 subjects (70%) that had either angular cheilitis or pruritic eruption had a CD4 cell count of <250cells/mm^3^. Thus 44 subjects would have been correctly considered eligible for ART if any of the two clinical features had been used, while only 19 of the 63 subjects would have been started on ART earlier than the required time if they were considered. The identification of 44 eligible patients using the supplemental clinical symptoms of angular cheilitis and papular pruritic eruption would have improved the sensitivity of the clinical staging from 53.5% to 73.0% and subsequently reduced the false negative rate from 46.5% to 27%.

To assess the effect of the use of angular cheilitis and recurrent upper respiratory tract infections on the sensitivity, specificity and false negative rate to predict CD4<350cells/mm^3^ among patients in Stage I and II, a variable “clinical factor present” was generated if any of the patients in Stage II disease had one or both clinical factors of angular cheilitis and recurrent upper respiratory tract infection present.

Sixty three (77.8%) of the 81 subjects that had either angular cheilitis or upper respiratory tract infections on both were found to have CD4 counts <350cells/mm^3^ and therefore would have been eligible for ART initiation, while only 18 of the 81 subjects would have been treated earlier than the required time if it they were considered. The identification of63 eligible subjects using only angular cheilitis and recurrent upper respiratory tract infections would have improved the sensitivity of the clinical staging from 49.1% to 71.5% and subsequently reduced the false negative rate from 50.9% to 28.5%.

## Discussion

Although the WHO HIV/AIDS clinical staging plays a critical role in guiding primary health workers in initiating antiretroviral therapy [3], [12], [13], some studies have reported diagnostic limitations of its use [8], [14], [15]. Our study findings show a sensitivity of 53.5% and 49.1% for identifying CD4 counts of less than 250cells/mm^3^ and 350cells/mm^3^, respectively. Our findings replicate the low sensitivities that have been previously found in studies of the WHO staging guidelines conducted in Africa [8], [14], [15]. The use of cotrimoxazole prophylaxis to prevent some opportunistic infections may have contributed to the high false negative rate, despite the low CD4 counts. The low sensitivities that were found show that about half of the participants would have missed ART initiation if the WHO clinical staging guidelines alone were used, which suggests significant diagnostic challenges to the WHO/HIV AIDS clinical staging guidelines which if used alone may impact morbidity and mortality [16], [17].

We found that the specificity of the WHO HIV/AIDS clinical staging guidelines were at 81.1% and 86.8% at CD4 counts of 250cells/mm^3^ and 350cells/mm^3^ respectively, which was also similar to previous studies using various CD4 cell count cut-offs [8], [9], [14]. Our results showed positive predictive values at CD4<250cells/mm^3^ and at CD4<350cells/mm^3^ of 79.1% and at 90.2%, respectively; and negative predictive values of 56.6% and 40.9% respectively.

The finding that females had a higher mean CD4 cell count than the males is similar to previous findings that demonstrated that women in stage 1 were less likely to have a lower CD4 cell count compared to the men [18]. This may be due to the better health seeking behaviour among women.

The urban based population in this study may have had a higher mean CD4 cell count compared to the rural population because of better access to the HIV counselling and testing services leading to earlier HIV diagnosis.

In our study, we found that angular cheilitis and papular pruritic eruptions were significant predictors of CD4 cell count <250cells/mm^3^ while angular cheilitis and recurrent upper respiratory tract infections predicted a CD4 cell count <350 cells/mm^3^. These results are in agreement with a Tanzanian study that found that HIV associated mucocutaeous manifestations may improve the sensitivity of the WHO staging guidelines [19]. The use of three clinical features, of angular cheilitis, papular pruritic eruption and upper respiratory tract infections might greatly improve the sensitivity of the WHO HIV/AIDS guidelines in identifying patients with WHO clinical stages 1 and 2 that are eligible to start treatment. This is the first study to demonstrate the utility of the angular cheilitis, papular pruritic eruptions and upper respiratory tract infections in identifying HIV patients with low CD4 cell count which would improve on the sensitivity of the clinical staging guidelines.

While the WHO HIV/AIDS clinical guidelines are an affordable method to determine ART eligibility, routine or low cost CD4 T-cell count, as compared with WHO HIV/AIDS clinical staging in a resource limited setting is very cost-effective for sub Saharan Africa [20].

However, the availability of the tests and the laboratory personnel is still limited in Uganda. Therefore, determining other clinical factors that are predictive of low CD4 cell count in clinical stages I and II, will be very useful in improving the identification of subjects eligible for ART initiation until low cost CD4 cell count testing is widely available in Uganda. We recommend that similar studies with larger sample sizes be conducted in other developing countries to assess the role of clinical predictors, in addition to WHO staging guidelines in determining equivalents to CD4 counts.

Our study had much strength; there was minimal referral bias since this was a multi centre study. Random error was minimised by having an adequate sample size for the common clinical predictors. The study was limited by a lack of sufficient sample size to detect the association between rare clinical conditions and CD4 counts below 250cells/mm^3^ and 350cells/mm^3^. Further studies are recommended to assess the association between rare clinical features and CD4 cell counts.

WHO HIV/AIDS clinical stage misclassification was minimised by use of experienced clinicians trained in the study protocols and blinding of the clinicians to the CD4 results.

Based on the findings of this study, we recommend increased access to CD4 cell count testing machines in resource limited settings. In the interim, more study is needed to confirm the clinical features that we found to be predictive of low CD4 counts. If these results are confirmed, then ART initiation among persons with these clinical features should be started regardless of WHO HIV/AIDS clinical stage. This will go a long way in identifying more patients eligible for ART.
